# Morphologic and genic effects of waste pollution on the reproductive physiology of *Paracentrotus lividus* lmk: a mesocosm experiment

**DOI:** 10.3389/fphys.2023.1161852

**Published:** 2023-05-23

**Authors:** Francesca Glaviano, Serena Federico, Bruno Pinto, Maissa Gharbi, Tania Russo, Anna Di Cosmo, Gianluca Polese, Maria Costantini, Valerio Zupo

**Affiliations:** ^1^ Stazione Zoologica Anton Dohrn, Department of Ecosustainable Marine Biotechnology, Ischia Marine Centre, Naples, Italy; ^2^ Department of Biology, University of Naples Federico II, Complesso Universitario di Monte Sant’Angelo, Naples, Italy; ^3^ Stazione Zoologica Anton Dohrn, Department of Ecosustainable Marine Biotechnology, Napoli, Italy

**Keywords:** dissolved organic compounds, recirculated aquaculture system, gene expression, larvae, sea urchin

## Abstract

A considerable amount of coastal contamination is caused by wastes deriving from household and the degradation and the metabolism of plants and animals, even if our attention is commonly focused on industrial pollutants and contaminants. Waste pollutants are mainly represented by highly diluted soluble compounds and particles deriving from dead organisms. This complex combination, consisting of suspended particles and dissolved nutrients, has a significant impact on coastal planktonic and benthic organisms, also playing an active role in the global cycles of carbon. In addition, production practices are nowadays shifting towards recirculated aquaculture systems (RAS) and the genic responses of target organisms to the pollution deriving from animal metabolism are still scarcely addressed by scientific investigations. The reservoir of organic matter dissolved in the seawater is by far the least understood if compared to that on land, cause only a few compounds have been identified and their impacts on animals and plants are poorly understood. The tendency of these compounds to concentrate at interfaces facilitates the absorption of dissolved organic compound (DOC) onto suspended particles. Some DOC components are chemically combined with dissolved metals and form complexes, affecting the chemical properties of the seawater and the life of the coastal biota. In this research, we compared the reproductive performances of the common sea urchin *Paracentrotus lividus* cultured in open-cycle tanks to those cultured in a recirculating aquaculture system (RAS), where pollution progressively increased during the experiment due to animal escretions. Sea urchins were cultured for 7 months under these two conditions and their gametes were collected. Embryos resulting by *in vitro* fertilization were analyzed by *Real Time qPCR* to identify possible effects of pollution-induced stress. The fertility of sea urchins was evaluated, as well as the gonadosomatic indices and the histological features of gonads. Our results indicate that pollution due to excess of nutrients, event at sub-lethal concentrations, may hardly impact the reproductive potential of this key species and that chronic effects of stress are revealed by the analyses of survival rates and gene expression.

## 1 Introduction

Global concern about the possible negative impacts of pollutants on ecosystems and humans has extensively increased in the last decades. Thousands of pollutants, most of which are organic (Bhomick et al., 2017) or deriving from metabolic wastes, have entered the environment because of such human activities as industrialization, agriculture and coastal urbanization (Daughton, 2005; Dachs and Méjanelle, 2010; Abbasi and Kamalan, 2018). Indeed, coastal areas, including transitional waters, are subject to considerable human influences on a global scale (Landrigan et al., 2020; Manisalidis et al., 2020; Asim et al., 2021; Ghazizade et al., 2021), because a major proportion of the world’s population historically resides in regions near the water bodies, inducing increased anthropogenic stresses to coastal ecosystems (Muir and Howard, 2006; Steffen et al., 2007). Even if the effects of several contaminants on aquatic organisms are well-known, the short-term consequences of excess of nutrients released into the marine environment received insufficient attention (Asher et al., 2007; Grzybowski et al., 2009). Organic pollutants deriving from the degradation of wastes (Li et al., 2021; Zhou et al., 2022), in coastal areas and elsewhere, have not been thoroughly investigated, partially due to analytical limitations and the limited cooperation among different scientific fields, such as environmental and analytical chemistry, marine biology and oceanography (Asher et al., 2007; Valiela, 2016). An undetermined number of chemicals have potentially been released into the environment, but they have still not been acknowledged in the scientific literature (Gigliotti et al., 2005; Jurado et al., 2005; Asher et al., 2007). In addition, the classical methods of investigation only permitted the identification of a small number of organic contaminants, using costly and time-consuming procedures (Valiela, 2016). Nowadays, due to huge advancements in analytical instruments, hundreds of emerging contaminants were identified up to trace levels in the last decades, tracking their actual accumulation. Several compounds deriving from a variety of applications, including drugs and cosmetics, agricultural herbicides, combustibles, detergents, aquaculture effluents and others, are constantly introduced into the marine environment (Gibbs, 1993; Muir and Howard, 2006).

In addition, it has been stated that aquaculture practices locally affect the aquatic surroundings (Aubin et al., 2006). The inorganic nutrient accumulation (mainly nitrogen and phosphorus) leads to eutrophication, and an increase in organic wastes in the ecosystems produces various negative effects both to the aquatic biota and to the same organisms cultured in RAS (recirculating aquaculture systems) tanks (Cole et al., 2009). Taken together, these issues and the pollution can lead to oxygen depletion, alteration of the water quality, decline of aquatic communities, algal blooms, mass mortality due to eutrophication and habitat loss (Boesch and Paul, 2001). Pollutants deriving from the degradation of wastes can access the coastlines through a variety of pathways, and they enter the biogeochemical cycles by sinking, and through bioaccumulation processes, after their first introduction (Gigliotti et al., 2005). Their environmental distribution strongly relies on the physical-chemical characteristics of the compounds, except in the proximity of source points. Once dead tissues and wastes reach the water, they flow until they are either decomposed, absorbed on sediments, or become sinking particles (Fleeger et al., 2003; Steffen et al., 2007).

The majority of ecotoxicology research have focused on evaluating the effects of single contaminants or simple combinations of pollutants (Muir & Howard, 2006). To mirror these realities, ecotoxicological investigations and approaches must undergo significant innovations (Jurado et al., 2005; Dueri et al., 2008), because several evidence indicate the direct and indirect effects of various pollutants on aquatic habitats, despite only 20% of previous studies focuses on oceans (Fleeger et al., 2003). In addition, aquaculture practices in RAS have been largely improved and the present trends of research are aimed at implementing new culture techniques in recirculating systems (Ahmed & Turchini, 2021), because of the urgent need to reduce the water consumption and the impacts of polluted wastes on marine coastal communities (Midilli et al., 2012). To this end, several species historically cultured in open-cycle tanks, or even in cages, are progressively transferred to RAS, in order to reduce the impacts, and in view of green aquaculture technologies (Gunning et al., 2016). This shift, however, also imposes to develop clear mind about the actual effects of pollutants normally increasing in RAS, when the density of cultured organisms is high and the limits of the life support systems (LSS) are met (Zohar et al., 2005). To this end, a good knowledge of the effects of various pollutants on the physiology of cultured organisms, along with their genic responses, becomes vital.

In this study, we employed the common sea urchin *P. lividus* as a model organism. This sea urchin represents an economically relevant species for the seafood market and a resource for scientific research. In addition, it plays a crucial role in the ecology of Mediterranean coastal ecosystems because it is one of the main grazers in algal and seagrass ecosystems. Consequently, increasing pollution events impacting shallow coastal ecosystems might influence its reproductive potential and the ecology of economically relevant communities. In addition, *P. lividus* is extensively adopted in embryological and developmental biology studies, and it is a perfect model organism for ecotoxicological and physiological surveys, thanks to its easy management in the laboratory and the transparency of embryos, which permits to follow the early stages of development. Embryotoxicity tests on sea urchin embryos can be rapidly completed (currently, at 24, 48, and 72 h (Matranga et al., 2010); on a huge number of individuals at the same time (Bonaventura et al., 2005), and the effects on embryonic differentiation can be observed both at the morphological level and the molecular level (Roccheri et al., 2004; Pinsino et al., 2011). Moreover, while the planktonic larval stage represents a useful indicator for short-term events, the settled individuals of *P. lividus* can be indicators for long-term phenomena.

Here, we investigated the effects of waters contaminated with waste pollution on the sea urchin *P. lividus* at morphological and molecular levels, by adopting a mesocosm experiment. In particular, adult sea urchins were cultured for 7 months in open-cycle tanks and compared to individuals cultured in a recirculating aquaculture system (RAS), where waste pollution progressively increased during the experiment, in the absence of water changes. The reproductive performances of sea urchins in these two conditions were analysed, as well as the gonadosomatic indices and the gonadic state, investigated by means of histological techniques. The expression levels of seventy-nine genes involved in the stress response, as well as in development/differentiation processes (such as those involved in the skeletogenesis) were evaluated by *Real Time* qPCR, to identify the functional pathways affected by pollutants progressively increasing in the culture tanks.

## 2 Material and methods

### 2.1 Ethics statement

Adult individuals of *P. lividus* (Lamarck) were collected in the Bay of Naples at a site that is not privately owned or protected, according to the Italian laws (DPR 1639/68, 19 September 1980, confirmed on 10 January 2000). Field studies did not include endangered or protected species. All experimental procedures on animals were in compliance with the guidelines of the European Union (directive 2010/63/EU).

### 2.2 Experimental set-up

Adult sea urchins, *P. lividus,* were collected by scuba divers in the Gulf of Napoli (Italy) and carried to the laboratory in thermostatic bags, to avoid stressing increases of temperature. They were gradually acclimatized in open-cycle tanks for 2 weeks prior to start the experiment, that was carried out for 7 months in four adjacent circular tanks. The diameter of tanks was 90 cm on average and the height was 66 cm (until the surface water level). Each tank was filled with 405 L of filtered saltwater, previously pumped from a pipeline located off the harbour of the Procida Island (about 60 m off the seashore). Two tanks were set as a Recirculating Aquaculture System (RAS), where waste pollution progressively increased during the experiment. Each RAS tank was equipped with an external mechanical filter (Whale, SICCE, Italy) and a skimmer (Seachem Aquavitro, Italy). In addition, the RAS tanks were equipped with a set of five submersible smart pumps XStream SDC (SICCE, Italy) mounted on the inner walls of the tanks through magnetic supports coated with protective gum, to dissipate vibrations. The pumps were managed by the smartphone app *Contrall* (Apple store and Google Play store), in order to become smart devices connected through Wi-Fi networks. The app *Contrall* provided real-time feedback on the status of the pumps and an alarm system which was activated in case of anomalies. The pumps in the upper part of the tanks were positioned in counter-clockwise and upward direction, and three pumps in the lower side were positioned in clockwise and downwards direction. These settings allowed to create two different and contrary currents that mixed and oxygenated the water. The pumps were also connected to an ORP probe, constantly measuring the value in each tank. The ORP controller permitted to set an ORP lower limit and, when the probe read values under a threshold, the pumps were activated mixing and aerating the water until the ORP values reached values above the set threshold.

The two control tanks, in their turn, were managed in open cycle conditions, to guarantee a continuous exchange of seawater. They continuously received clean seawater pumped from a pipeline set off the harbour of the Procida Island (Bay of Naples), filtered into a large sump connected to a protein skimmer, and finally directed into a distribution pipeline reaching the tanks. The output water was re-directed to the sump, where an overflow permitted to wash it out in the harbour of Procida. Twelve complete water changes per day (every 2 h) were assured by the water pumped into the open cycle system. An aeration device was also set inside each experimental and each control tank, to maintain dissolved oxygen (DO) at healthy levels for sea urchins and guarantee water circulation in the tanks.

After the complete setup, thirty sea urchins (*P. lividus*) were added to each tank (both test tanks and control tanks), i.e., 20 females and 10 males (female/male ratio of 2:1). The sex of sea urchins was previously determined under an optical microscope, based on the dimorphism in terms of shape and size of five dermal plaques visible around the anus (Brundu et al., 2022) During the experiment, the sea urchins reared in RAS tanks and those in the control tanks were fed twice a week *ad libitum* on a highly proteic pellet (Greenvet, Italy). The main water parameters, namely, temperature, dissolved oxygen, redox potential, salinity and pH were checked manually three times a week. Nitrites, nitrates, phosphates and ammonia concentrations were checked using a colorimetric test (by adopting standard analytical kits for the photometer AL450, Aqualytic, Germany). The above-mentioned data measured in RAS tanks were compared to those measured in open cycle tanks.

### 2.3 Biotic and abiotic variables

Physical and chemical indicators of the seawater quality were measured every 3 days in each tank. Water samples were collected in 50 mL beakers from the tanks to be analysed by the filter photometer AL450 (Aqualytic, Germany). Chemical analyses measured the concentration (ppm) of nitrites, nitrates, ammonia and phosphates, as easy indicators of waste pollution. Physical variables were also monitored. Temperature was daily recorded at noon by an alcohol thermometer; salinity was measured by means of a refractometer (TTBH Pte Ltd., Singapore); dissolved O_2_ was measured by means of an Oxygen portable meter (ProfiLine oxi 3,310, WTW, Germany); pH was measured by a multiparametric probe (XS Instruments®, PC 7 Vio, Italy). In addition, behaviour, spawning, mortality and the health status of sea urchins were daily checked in the tanks and recorded in a spreadsheet.

### 2.4 *In vitro* fertilization for morphological and molecular analyses

Five sea urchins of each gender were injected with 1 mL of 0.5 M KCl through the peristomal membrane, to stimulate the contraction of gonads and to obtain the gametes. The subjects were then vigorously shaken and females were placed with their mouths up, over 50 mL beakers, until the gametes were released into filtered (0.22 μm Millipore) seawater, to facilitate the collection of oocytes, which were rinsed three times with clean seawater to remove possible organic residuals. Sperms were collected dry from the gonophores to avoid premature activation that takes place when the sperms remain in direct contact with seawater. The eggs obtained were pooled in Petri dishes (diameter 14 cm) filled with filtered seawater. Embryos were incubated in a thermostatic chamber at 18°C for 48 h until reaching of the *pluteus* stage; subsequently larvae were fixed in glutaraldehyde (4%) and observed under an optical microscope to evaluate the percentage of malformations, according to (Mcedward, 1984; Pagano et al., 1986). The significance of differences was determined by means of t-tests.

Fourthy-eight hours post-fertilization (hpf), about 5,000 fertilised eggs were collected from each of five females. The samples were centrifuged at 4°C for 15 min at 3,500 rpm. The embryos were then conserved in RNAlater (Qiagen, Hildesheim, Germany), frozen in liquid nitrogen and then stored at −80°C until use. Total RNA was extracted using *Aurum Total RNA Mini Kit* (BioRad, Hercules, California, United States). Using a NanoDrop spectrophotometer (ND1000 UVVIS Spectrophotometer; NanoDrop Technologies, Wilmington, DE, United States). The quantity of total RNA extracted was determined by the absorbance at 260 nm and the purity by the 260/280 and 260/230 nm ratios. To obtain cDNA, 1,000 ng of total RNA was retrotranscribed for each sample using an *iScript cDNA Synthesis kit* (BioRad, Milan, Italy). In addition, adults were weighed, sacrificed and dissected; their gonads were extracted and weighed (fresh weight) for the evaluation of the gonadic indices (GI%). The evaluations of the GI% were performed on all specimens in the test tanks as compared with all the specimens still present in the control tanks at the end of the experiment.

### 2.5 Variations of the gene expression

The variations in the expression of 27 genes involved in the stress response, 43 genes involved in development/differentiation processes, 8 genes involved into skeletogenesis and 9 in detoxification processes (see Supplementary Table S1 in the Supplementary Materials for their biological functions) were evaluated by *Real Time qPCR*. Undiluted cDNA was used as a template in a reaction containing a final concentration of 0.3 mM for each primer and 1 × FastStart SYBR Green master mix (total volume of 10 µL) (Applied Biosystems, Monza, Italy). PCR amplifications were performed *CFX96 Touch Real-Time PCR Detection System* (Bio-Rad Laboratories, Inc.), using the following thermal profile: 95°C for 10 min, one cycle for cDNA denaturation; 95°C for 15 s and 60°C for 1 min, 40 cycles for amplification; 72°C for 5 min, one cycle for final elongation; one cycle for melting curve analysis (from 60°C to 95°C) to verify the presence of a single product. Each assay included a no-template control for each primer pair. To capture intra-assay variability, all real-time qPCR reactions were carried out in triplicate.

For all *Real-Time* qPCR assays, the results of each cDNA sample were standardised with the mRNA level of the housekeeping genes *18S rRNA* and Cytochrome c oxidase used as reference genes, whose expression levels are rather stable throughout the development. Fluorescence was measured using *Bio-Rad CFX Maestro software* (Bio-Rad Laboratories, Inc.). The values of C (t) obtained and the efficiency values for each pair of oligonucleotides are analysed and normalised against the internal control by REST programme (*Relative Expression Software Tool*) based on the Pfaffl method, and the expression values of the gene of interest relative to the control were reported (Pfaffl, 2001; Pfaffl et al., 2002). Relative expression ratios greater than ±1.5 were considered significant.

### 2.6 Histological analyses

The gonads of one male and four females for each treatment were collected, fixed in Glutaraldehyde solution (4%), dehydrated in ascendant ethanol, clarified in methyl benzoate and included in paraffin according to Zupo et al. (2018). Five µm slices were obtained with a microtome (Leica Histocore Biocut) and stained with haematoxylin to detect any presence of morphological alterations. Histopathological indices were calculated according to Costa et al. (2013).

### 2.7 Data collection and statistical analyses

A Student *t-*test was applied to determine the significance of differences between data-sets whose normality of variance was previously tested by means of the Shapiro-Wilk test. In trials where a larger number of samples were compared, one-way ANOVA was adopted, to determine the significance of differences among experimental groups, after testing the normality and the homogeneity of variances by the D’Agostino and Pearson’s test. A matrix “parameters vs times of measure” also including mortality rates was built, and similarity matrices were obtained using a Pearson correlation coefficient, in order to check the relationships among variables, for all the considered datasets. Correlation matrix analyses were used to display the Pearson correlation coefficients (a measure of the linear association between two variables) among the seawater parameters and mortality events in the tanks. The significance of correlation patterns was evaluated using the confidence intervals of the Spearman correlation indices. This simple analysis permitted an immediate evaluation of relationships among abiotic parameters, as well as the identification of variables best related to the mortality rates.

For the evaluation of the GI%, sea urchins were weighed, sacrificed and dissected; their gonads were extracted and weighed (fresh weight) and the index was calculated according to the formula proposed by Fabbrocini and D’Adamo (2010) and Keshavarz et al. (2017):

1) GI = gonadal wet weight (g)/sea urchin wet weight (g) × 100

Histopathological indices were calculated using the formula proposed by Costa et al. (2013):

$$I_{h}=\frac{\sum_{1}^{j}w_{j}a_{jh}}{\sum_{1}^{j}M_{j}}$$

where, *Ih* is the histopathological index for the individual *h*; *wj* is the weight of the *j*th histopathological alteration; *ajh* is the score attributed to the *h*th individual for the *j*th alteration and *Mj* is the maximum attributable value for the *j*th alteration (if all alterations are present at the maximum diffusion). The *Ih* was determined following the concepts of the differential biological significance of each analysed alteration (weight) and its diffusion (score). The weights ranged from 1 (minimum severity) to 3 (maximum severity), while the score varied from 0 (not present) to 6 (diffuse). As histopathological alterations, we considered: the presence of lipofuscin (w = 1); cells in atresia (w = 2); cells in necrosis (w = 3). The diffusion was calculated using the presence/absence of each alteration in 6 random pictures taken at magnification of ×40 for each specimen. A PERMANOVA test was performed to determine the significance of differences in the *Ih* index among all treatments. All graphs and statistical analyses were processed using GraphPad Prism 8.0 (GraphPad Software, San Diego, California United States, www.graphpad.com).

## 3 Results

### 3.1 Seawater chemistry

**FIGURE 1:**
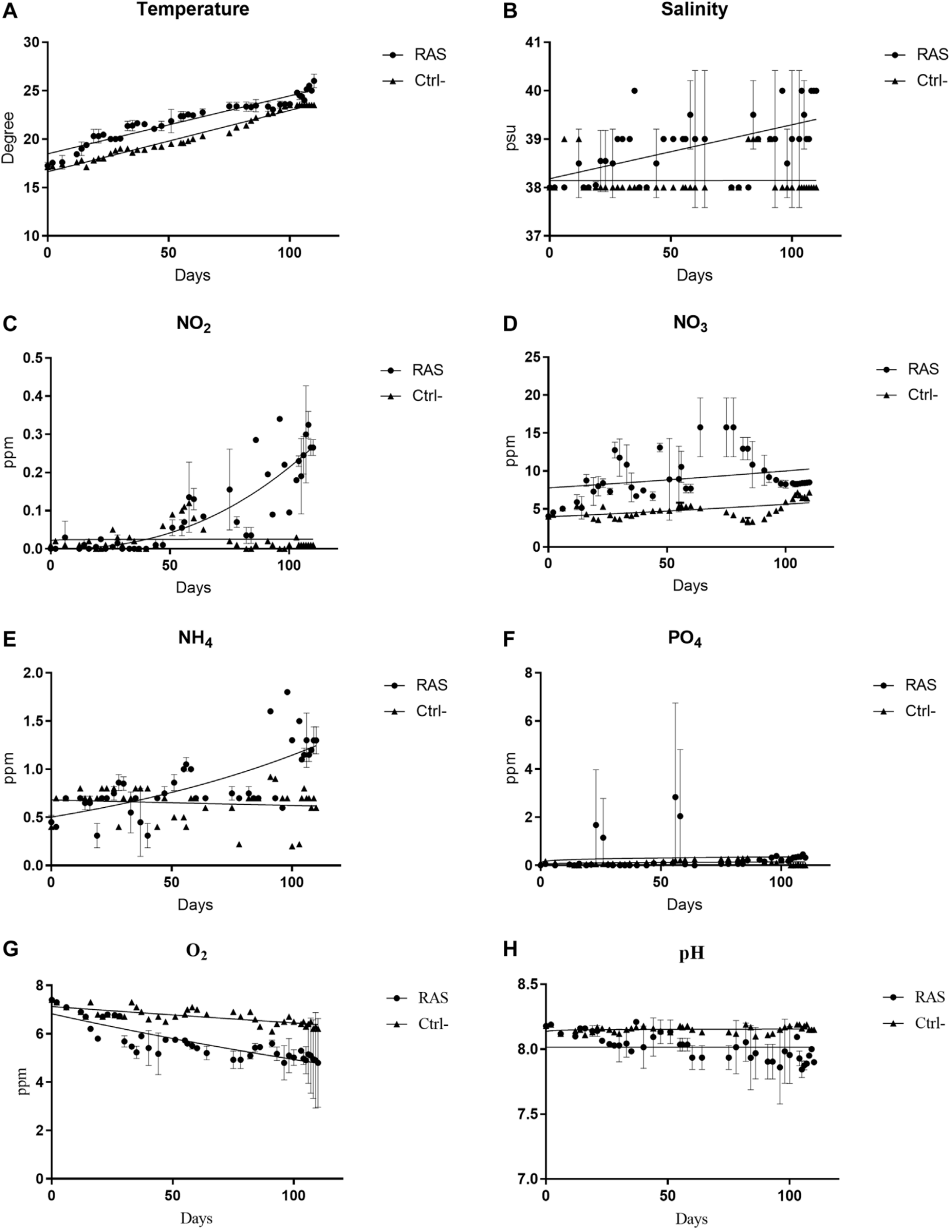
Chemical and physical water trends in RAS tanks and control (Ctrl) tanks. **(A)** Temperature; **(B)** Salinity; **(C)** Nitrite (NO_2_); **(D)** Nitrate (NO_3_); **(E)** Ammonia (NH_4_); **(F)** Phosphate (PO_4_); **(G)** dissolved oxygen; **(H)** pH.

The concentration of NH_4_ in the water of both RAS tanks (test tanks), ranged between 0 and 1.8 ppm (a maximum at 1.8 ppm was recorded at the end of the experiment). Taking into account the mean weekly concentrations of NH_4_ (Figure 1E), similar time trends were revealed in both RAS tanks. In the control tanks (open cycle) the concentration of NH_4_ exhibited a gradual increase from the 60th day until a stable concentration of 0.9 ppm in the last days of the experiment (Figure 1E). The trends of ammonium concentration significantly differed in experimental tanks, with respect to the control tanks (*p* < 0.05; Supplementary Table S2). The concentration of NO_2_ in the RAS tanks ranged between 0 and 0.39 while in control tanks (Figure 1C) it ranged between 0 and 0.12. The Student *t*-test showed a significant difference between RAS tanks and the control tanks, (*p* < 0.05; Supplementary Table S3). The concentrations of NO_3_ in the RAS tanks exhibited very irregular trends and ranged between 4.1 and 18.5. The weekly average of NO_3_ concentrations indicated variable trends in the RAS tanks, and significant differences with respect to the control tanks (Figure 1D). The nitrate concentrations in control tanks ranged between 3.1 and 7.3. Thus, nitrates were kept consistently lower than in the RAS tanks (Figure 1D) (*p* < 0.05; Supplementary Table S4). The concentration of PO_4_ ranged between 0 and 5.6 in the RAS tank 1 and between 0 and 0.45 in RAS tank 2 (Figure 1F). The mean concentration of phosphates in control tanks ranged between 0 and 0.3, and no significant differences were revealed between the RAS and the control tanks (*p* > 0.05; Supplementary Table S5). The temperature in the RAS tanks varied along with external temperature, continuously increasing from March to August (Figure 1A). In the control tanks the temperature trends were characterized by a gradual increase, but the maximum was 23.5°C, was reached in the last period. The Student *t-*test indicated significant temperature differences (*p* < 0.05; Supplementary Table S6) between control and test tanks. The pH ranged between 7.8 and 8.2 in the RAS tanks (Figure 1H) while it was significantly different in control tanks (*p* < 0.05; Supplementary Table S7), where it was consistently above 8. The concentration of dissolved O_2_ was between 3.5 and 7.4 ppm in RAS tanks, while in the control tanks it was significantly different (*p* < 0.05; Supplementary Table S8) and ranged between 6.1 and 7.4 ppm (Figure 1G). The salinity of water ranged between 38 PSU and 40 PSU in RAS tanks, while in control tanks it significantly differed (*p* < 0.05; Supplementary Table S9) and remained stably around 38 PSU, with a few increases at 39 PSU (Figure 1B).

### 3.2 Survival rates and water conditions

**FIGURE 2:**
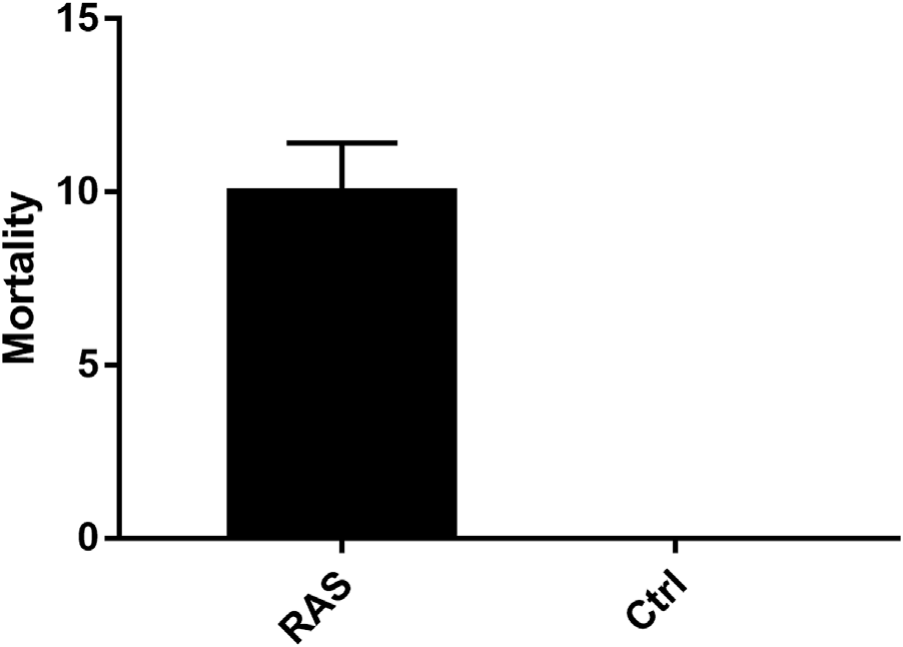
Mortality rates of animals reared in the RAS tanks and control tanks.

Significant differences in mortality rates were detected between the control tanks (where no mortality was detected at all) and RAS tanks (*p* < 0.05; Supplementary Table S10; Figure 2). The water descriptors in RAS tank 1 and 2, analysed by means of correlation matrices, indicated a significant relationship between sea urchin mortality and records of water quality out of their optimal ranges for *P. lividus*. In particular, a significant difference between RAS systems and the control tanks was detected in all physical and chemical descriptors of water in the last days of the experiment, when an increase of mortality was recorded, especially in the last days (Figure 1).

**FIGURE 3:**
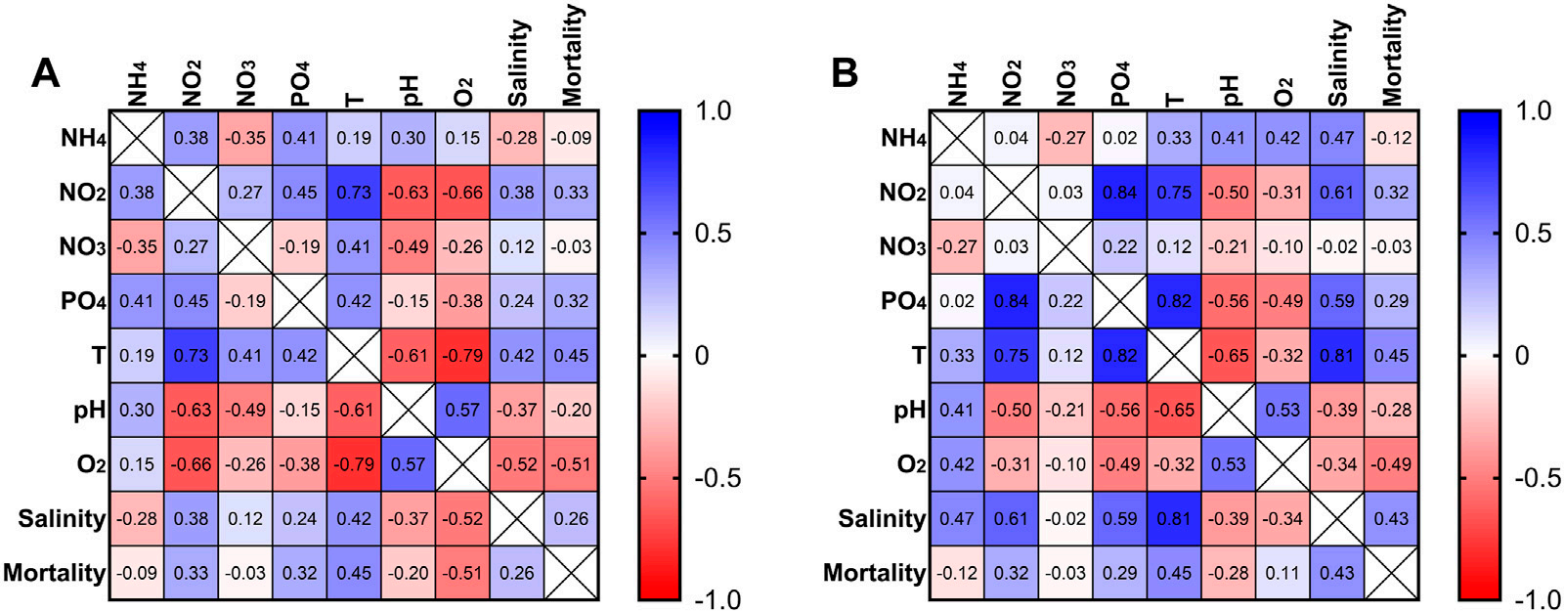
Correlation matrix for different measures from RAS tank 1 **(A)**. Correlation matrix for different measures from RAS tank 2 **(B)**.

The correlation matrix (Figure 3A) for the RAS tank 1 showed that NH_4_ was negatively correlated (−0.35) with NO_3_, and positively correlated (0.41) with PO_4_. NO_2_ exhibited a moderate positive correlation (0.45) with PO_4_. As well, NO_3_ was positively correlated with temperature (0.41), and negatively with the pH (−0.49). PO_4_ was positively correlated (0.42) with temperature. The pH exhibited a positive correlation (0.57) with dissolved O_2_ since the two parameters increased in parallel. The dissolved oxygen exhibited a negative correlation with the sea urchin mortality (−0.51). A moderate negative correlation (−0.61) between temperature and pH was also observed, as well as a moderate positive correlation (0.42) with the salinity. However, temperature exhibited a moderate correlation (0.45) as well with sea urchin mortality in all the tanks and a negative correlation (−0.79) with the dissolved oxygen, indicating a strong decrease in the dissolved oxygen when the temperature increased.

In the RAS tank 2, the correlation matrix (Figure 3B) showed for NH_4_ a positive moderate correlation with temperature (0.33), while the correlation indices were 0.47 with salinity, 0.41 with pH and 0.42 with dissolved O_2_. NO_2_ exhibited a strong positive correlation (0.84) with PO_4_ and temperature (0.75). As well, a moderate positive correlation between NO_2_ and salinity (0.6) was indicated by this analysis. PO_4_ exhibited a strong correlation with temperature (0.82), as well as a moderate correlation with mortality (0.29) and salinity (0.59). Temperature showed a moderate negative correlation with pH (−0.65), while its correlation indices were 0.45 vs. mortality, and 0.81 vs. salinity. The pH showed a moderate correlation with the dissolved O_2_ (0.53). The dissolved oxygen exhibited a negative correlation with the mortality (−0.49). Increases of NO_2_ concentration corresponded to increases of PO_4_, and when also phosphates increased, mortality events were recorded. In contrast, the corelation matrices in control tanks did not indicate any relationship of the seawater descriptors with mortality events, because of a total absence of mortality.

### 3.3 Efficiency of *in-vitro* fertilization and gonado-somatic indices

**FIGURE 4:**
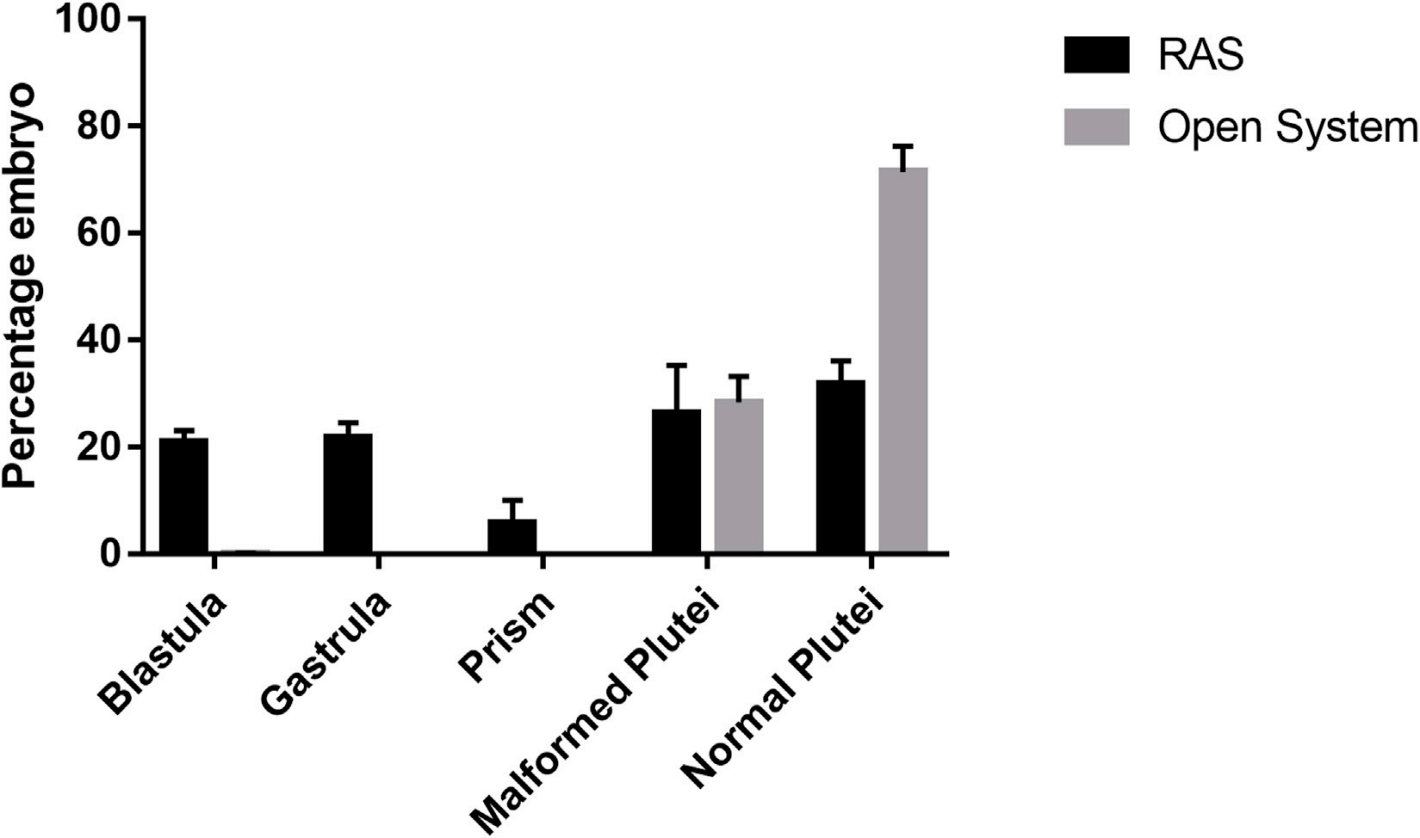
Percentage of malformed and delayed embryos at 48 h post-fertilization from sea urchins reared in RAS tanks and Open System.

**FIGURE 5:**
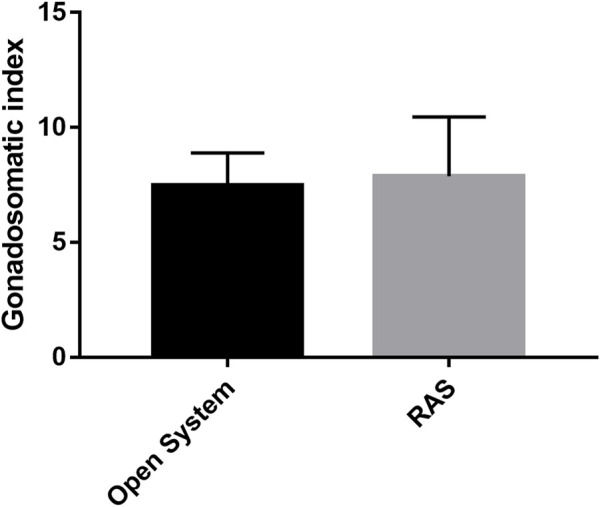
Gonadosomatic indices recorded at the end of the experimental period from sea urchins reared in RAS tanks and control tanks.

Fertilization rates ranging from 98% to 100% were observed using gametes deriving from animals from both RAS and control tanks (*p* > 0.05). In contrast, different results of larval development and malformations were observed between the two types of treatments. In fact, the tests in the RAS systems produced at 48 hpf: 20.93% (±2.23) of embryos still at the blastula stage; 21.75% (±2.76) of embryos still at the gastrula stage; 5.85% (±4.31) still at prism stage (Supplementary Table S11A; Supplementary Figure S1); 26.4% (±8.91) malformed plutei and only 31.85% (±4.3) of normal plutei (Figure 4A; Supplementary Table S11B). Inversely, the control tanks produced only 0.15% (±0.07) of embryos still at the blastula stage; 27.4% (±4.81; Supplementary Table S11A) of malformed plutei and 72.45% (±4.88) of normal plutei (Figure 4B). The differences between the two treatments were significant (*p* < 0.05; Supplementary Table S11B). Despite the significant differences in the production of healthy offspring, the adult sea urchins collected at the end of the experiment exhibited no significant differences in the gonadosomatic indices between the two different culturing systems (Figure 5; Supplementary Table S12).

### 3.4 Histological analyses

**FIGURE 6:**
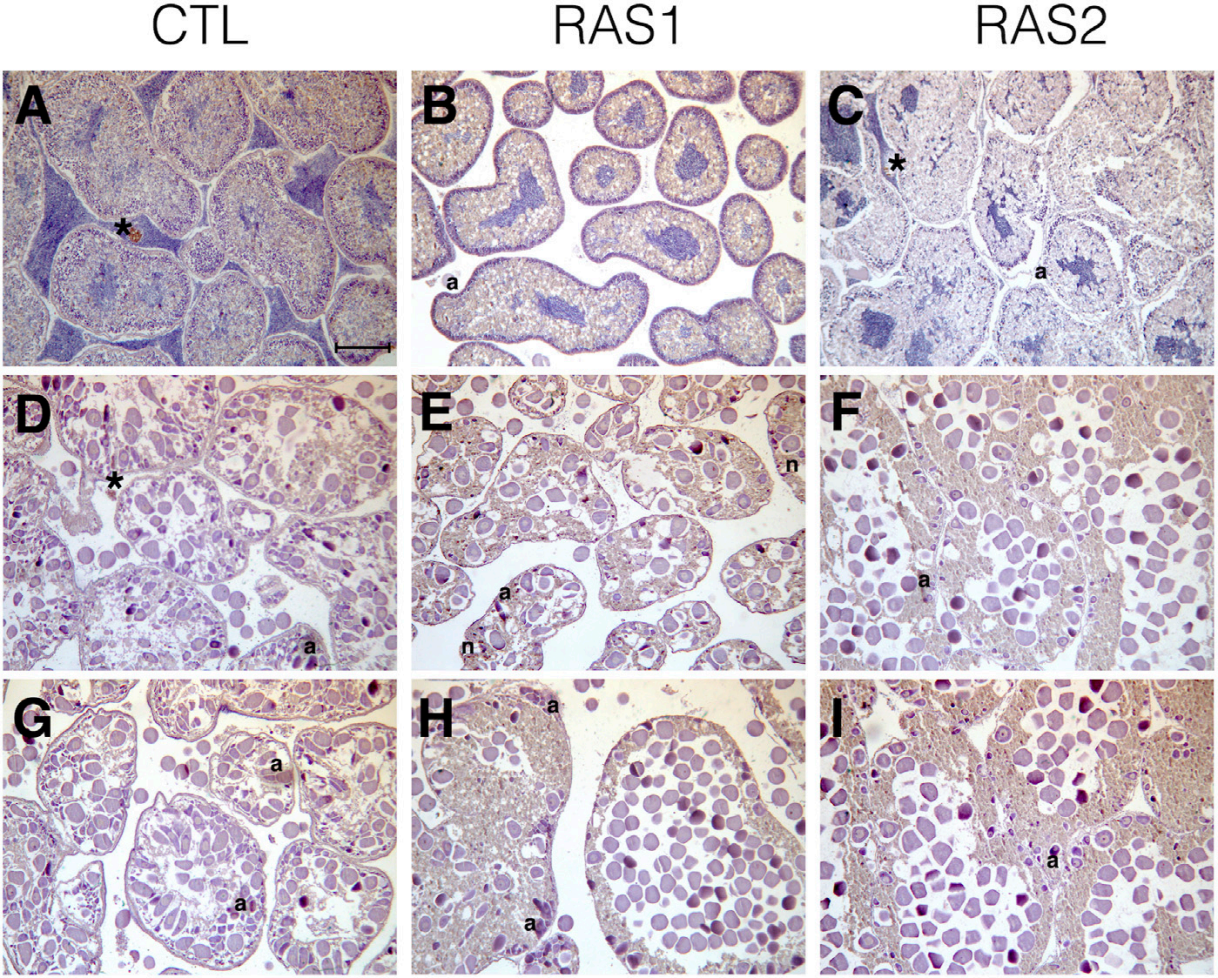
Overview of gonads sections from specimens raised with different water circuitry. **(A–C)**, testicles; **(D–I)** ovary sections; CTL, control system; (*lipofuscin aggregate, a atrasia, n necrosis) scale bar = 100 µm.

**FIGURE 7:**
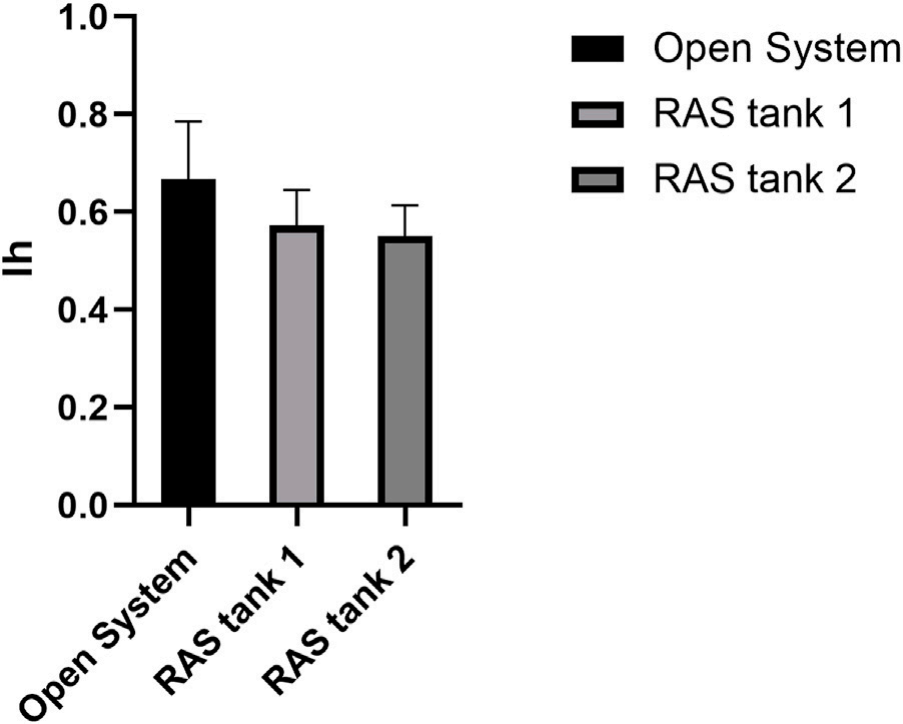
Ih: the graph shows the comparison of Ih among difference treatments, C = control tanks, V1 and V2 = RAS.

Histological analyses indicated that all tested specimens were at the stage of sexual maturity, with no substantial differences among the housing methods (Figure 6). The only noticeable differences between the open system and the RAS consisted in the absence of sperms in the interstitial spaces of the testis belonging to specimens cultivated in RAS (Figures 6A–C). The ovaries exhibited similar patterns in control and in RAS-reared specimens, but variable maturation levels were exhibited, with the presence of mature eggs in the interstitial gonad space (Figures 6D–I). The histopathological index, as well, did not reveal significant differences among treatments (Figure 7).

### 3.5 Variations of gene expression

#### 3.5.1 Stress genes

**FIGURE 8:**
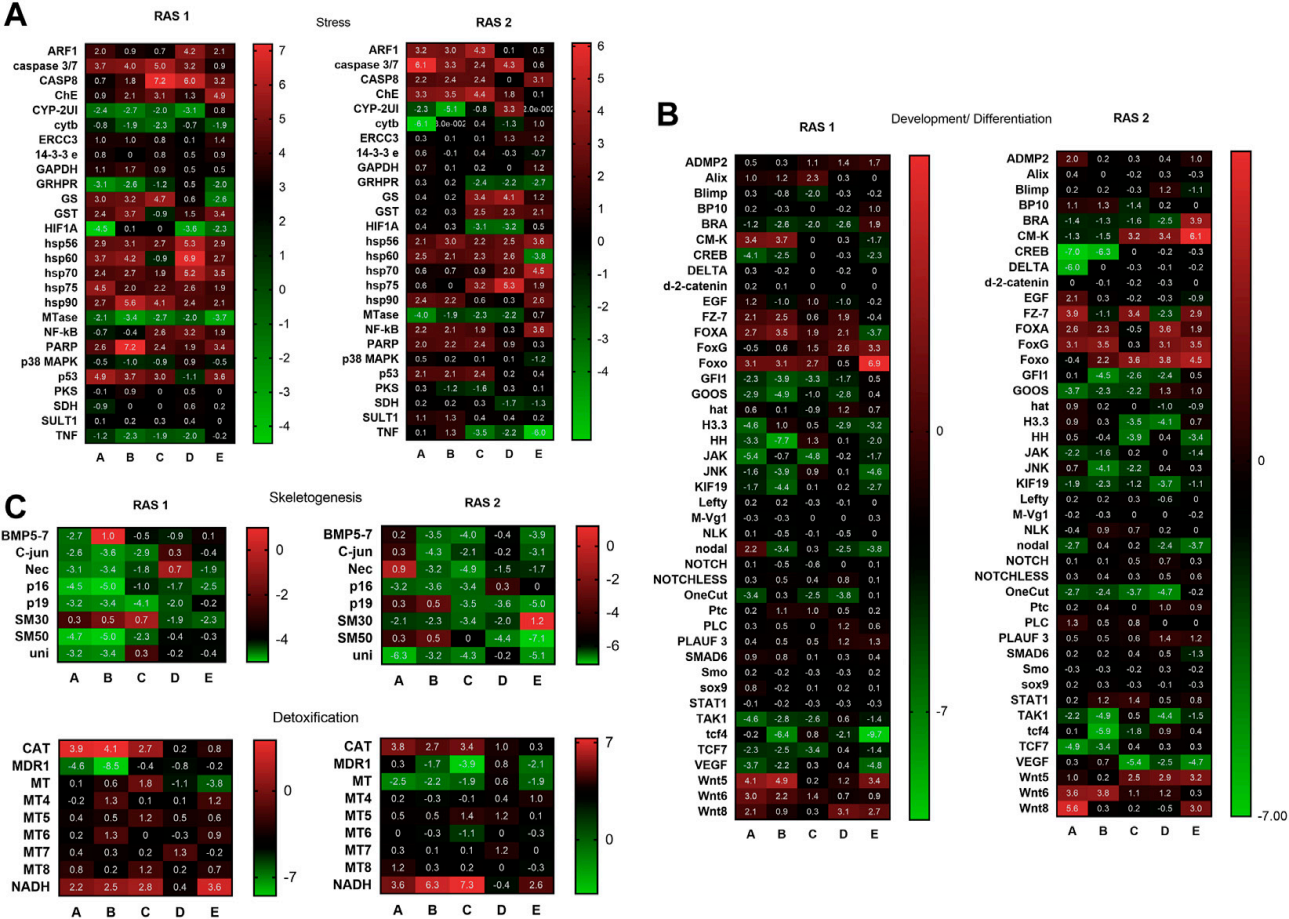
Heatmaps showing the expression profiles and hierarchical clustering of genes analysed by real-time qPCR in embryos deriving from *P. lividus* sea urchins reared in RAS tank 1 (on the left) and RAS tank 2 (on the right). **(A)** key genes involved in the stress response are shown; **(B)** key genes involved in skeletogenesis and detoxification pathways are shown; **(C)** key genes involved in development/differentiation are shown. A-B-C-D-E in the columns are the different replicates coming from different females. Colour code: red, upregulated genes with respect to the control; green, downregulated genes.

The variation of gene expression levels was followed by Real Time qPCR (Supplementary Table S1; Figure 8) for the genes of interest, belonging to four functional classes. The results referred to twenty-seven genes analysed showed that the plutei deriving from the RAS tanks had a significant variation in the expression for most of the genes analysed (Figure 8; Supplementary Table S1). In the case of plutei deriving from RAS tank 1, twelve genes increased their expression levels (Figure 8)*: caspase 3/7, CASP8, ChE, GS, GST, hsp56, hsp60, hsp70, hsp75, hsp90, NF-kB, PARP*, and *p53*. Six genes reduced their expression levels: *CYP-2UI, cytb, GRHPR, HIF1A, MTase*, and *TNF*. Similarly, in the case of plutei deriving from the RAS tank 2, fourteen genes increased their expression levels (Figure 8): ARF1, caspase 3/7, CASP8, *ChE, GS, GST, hsp56, hsp60, hsp70, hsp75, hsp90, NF-kB, PARP*, and *p53*. Six genes decreased their expression levels: *CYP-2UI, cytb, GRHPR, HIF1A, MTase* and *TNF*.

#### 3.5.2 Development and differentiation genes

Concerning the forty-five genes involved in the development and differentiation processes, the results showed that the plutei deriving from the RAS tanks have a further significant variation in expression for most of the genes analysed (Figure 8; Supplementary Table S14A). In the case of plutei deriving from RAS tank 1, six genes increased their expression levels: *CM-K*, *FOXA*, *FoxG*, *Foxo*, *Wnt5*, and *Wnt6*. Instead, sixteen genes decreased their expression levels: *BRA*, *CREB*, *FZ-7*, *GFI1*, *GOOS*, *H3.3*, *HH*, *JAK*, *JNK*, *KIF19*, *nodal*, *OneCut*, *TAK1*, *tcf4*, *TCF7*, and *VEGF*. Similarly, in the case of plutei deriving from RAS tank 2, eight genes decreased their expression levels (Supplementary Table S14B): *CM-K*, *FZ-7*, *FOXA*, *FoxG*, *Foxo*, *Wnt5*, *Wnt6*, and *Wnt8*. Conversely, sixteen genes decreased their expression levels: *BRA*, *CREB*, *FZ-7*, *GFI1*, *GOOS*, *H3.3*, *HH*, *JAK*, *JNK*, *KIF19*, *nodal*, *OneCut*, *TAK1*, *tcf4*, *TCF7*, and *VEGF*.

#### 3.5.3 Skeletogenic genes

The results referred to eight genes involved in skeletogenesis showed that the plutei deriving from the RAS tanks had consistently a significant expression variation for most genes analysed, resulting downregulated. In the case of plutei deriving from the RAS tank 1 (Figure 8; Supplementary Table S15A): *C-jun*, *Nec*, *p16*, *p19*, SM50 and *Uni* were downregulated. Similarly, in the case of plutei deriving from RAS tank 2 (Supplementary Table S15B): *BMP5-7*, *C-jun*, *Nec*, *p16*, *p19*, *SM30*, *SM50*, and *Uni* were downregulated.

#### 3.5.4 Detoxification genes

The genes involved in detoxification processes showed that the plutei deriving from the RAS tanks had an expression variation for some genes analysed. In the case of plutei deriving from RAS tank 1 (Figure 8; Supplementary Table S16A), *CA*, *MDR1*, and *NADH* exhibited significant variation of their expression. Similarly, in the case of plutei deriving from RAS tank 2 (Supplementary Table S16B), *CAT*, *MDR1*, *MT*, and *NADH* exhibited significant variation.

## 4 Discussion

Conventional ecotoxicity tests enable the identification of one or a few more substances, which can have a major harmful effect on organisms. The benefits of such methods are evident, because they may provide important information on the impacts of single pollutants on the physiology of a model species, but they do not consider the impact of a mixture of natural pollutants, as they are normally co-present in the environment. Consequently, an implementation of methods enabling fully understanding of the impacts of complex combinations of contaminants is required. This may be accomplished by concentrating the seawater and testing the physiological responses of individuals and communities employing a more realistic mesocosm. Such a kind of investigations could enable significant progresses in understanding how known and unknown contaminants impact coastal ecosystems and communities (Dachs and Méjanelle, 2010).

In our experimental set-up, despite the presence of the skimmers, pumps and the external filters and their constant maintenance, the increase in nutrients and waste-derived substances, as well as stochastic mortality events, induced gradual alteration of the water quality. As a result, the abiotic and biotic descriptors showed significant variations within a larger range in RAS tanks, as compared to those measured in the control tanks. Considering the high organic loads and the low dilution rates, nitrites, nitrates, phosphates, temperature and salinity might, as expected, accumulated in RAS systems more than in the controls, reaching higher concentrations, while pH and DO were lower.

The most relevant difference between the two systems was represented by a total absence of mortality in the control tanks, revealing good health conditions of animals in the open system, while mortality events occurred, often contemporaneously, in the RAS tanks. The correlation matrix obtained from the data collected in RAS tanks indicated that, rather than a single descriptor or factor, the continuous exposure and the additive effects over time led to stress responses and, consequently, to death. However, their effects may still be considered “sub-lethal,” since most reared individuals reached the end of the experiment. The increase of temperature and dissolved oxygen have already been identified as critical factors in aquaculture in general (Qiang et al., 2019) and in echinoculture in particular (Siikavuopio et al., 2007). For this reason and according to the correlation matrices, we can state that the increase in temperature and the decrease of oxygen strongly affected the general quality of the water and the health state of the sea urchins, leading to mortality events in both RAS tanks. The volume of water in the RAS tanks was limited and, without any water changes, its quality was strongly influenced by high temperature, which in turn could amplify pollution effects. In fact, this evidence was well supported by other water quality descriptors, which were strictly correlated with both variables. Accordingly, the temperature increases also induced an increase of salinity and PO_4_ concentrations. The increase of salinity was moderately correlated with the decrease of DO in the RAS tank 1, while in the RAS tank 2 the salinity was moderately correlated with the increase of NH_4_, NO_2_, and PO_4_. Considering its ecological adaptations, *P. lividus* has a considerable tolerance for salinity variations (Santos et al., 2022), but salinity increases can also lead to alteration in the microbial community influencing nitrification and denitrification processes (Xia et al., 2019). Dissolved oxygen is a crucial variable as well. It not only directly affects the health of sea urchins (Siikavuopio et al., 2007), but it is also fundamental for the decomposition of toxic substances (Zhang et al., 2020). Moreover, low levels of dissolved oxygen can limit nitrification and lead to the increase in CO_2_ content, and consequently to the decrease of the pH value. In both RAS tanks, dissolved oxygen presented a negative correlation with mortality, indicating that the water quality was highly altered by a low concentration of dissolved oxygen. In fact, dissolved oxygen in aquaculture drops significantly when temperature and density organic matter increase (Zang et al., 2011). These variations were not recorded in the control tanks, where the water was continuously renewed.

Sea urchins are known for being very sensitive to environmental fluctuations (Fernandez and Boudouresque, 1997), which can affect their reproductive cycle and lead to fertility decreases, or abnormal larval developmental (Byrne et al., 2011; Perez and Lehner, 2019). Subsequently, in this study we reported how the progressive increase of organic pollution can affect the reproduction success of *P. lividus*. Interestingly, the different culture conditions did not affect sea urchin gonad growth. In fact, at the end of the experimental period, the gonadosomatic index exhibited no significant differences among animals reared in the RAS system and in the control system. Sea urchin gonads are considered as structural storage tissue; the reserve takes place both through gonad increase in size or lipids and carbohydrates accumulation (Klinger et al., 1988; Fernandez, 1997). Generally, nutrient storage and gonadic development in *P. lividus* (Boudouresque and Verlaque, 2001) and other echinoderms (Barker et al., 1998) were considered mostly linked to food quality and availability, instead of the water temperature. For this reason, during the experimental period, all sea urchins were fed on the same diet *ad libitum*, to avoid any effect on the physiology of gonads. Nevertheless, gametogenesis can be affected by water temperature (Shpigel et al., 2004). Our histological analyses confirmed that all the adults tested (both males and females) achieved a sexual maturity stage, with no substantial differences. On the other hand, the results herein obtained from both the morphological observations on the larvae and the molecular analyses, indicate that an increased organic pollution may hardly impact the reproductive potential of this species. In fact, a significant difference was shown between the percentage of malformed plutei at 48 h, deriving from gametes produced by animals reared in the RAS system. Furthermore, our findings showed that more than 50% of embryos were delayed, being 48 hpf at the blastula or gastrula stage. In addition, some of them exhibited evident apoptotic changes (see Supplementary Figure S1). These results evidenced a strong effect on *P. lividus* reproductive efficiency. These morphological observations are well supported by the evaluation of the expression of several genes, isolated form the *P. lividus* transcriptome, related to different functional processes as stress response, development, differentiation, skeletogenesis and detoxification (see Supplementary Table S1 for gene functions). An important point to consider concerns the fact that the genes analyzed were functionally intercorrelated (Marrone et al., 2012; Varrella et al., 2016; Ruocco et al., 2017; Esposito et al., 2020), so to offering a clear and complete picture of the response to the DOCs by the sea urchin. Almost all genes under analysis switched on, as compared to the controls. Most of the genes involved in development and skeletogenesis were downregulated, justifying the morphological low success of embryonic development observed. In addition, it is evident that sea urchins exposed to these treatments attempted to detoxify, by increasing the expression of the specific genes involved in detoxification pathways. These findings indicated that the prolonged exposure of sea urchins to organic pollution, even if not inhibiting their gonadal maturation, was sufficient to affect common molecular pathways, altering some physiological mechanisms, which in turn can lead to morphological malformations in their offspring. Reproductive success is evidently fundamental for the survival of any species. Failure of adult sea urchins to produce embryos able to correctly develop, might induce strong impacts on their natural stocks.

Considering the limitations of standard ecotoxicology tests, the realistic mesocosm tested in this study can be considered as an effective method which, in combination with molecular analyses, helps our understanding of the impacts of complex combinations of stressors and accumulation of waste-derived contaminants in marine environments. In addition, the understanding of the effects of polluttants discharged from human activities can be extremely important to forecast and manage possible environmental damages associated with their rise and spread. Understanding the molecular processes involved in sensing and dealing with classical (waste-derived) or novel (industrial) contaminants might be useful to produce diagnostic tools to timely assess various threats to the marine environment.

## Data Availability

The original contributions presented in the study are included in the article/Supplementary Material, further inquiries can be directed to the corresponding authors.
