# Supplementary material for: Morphologic and genic effects of waste pollution on the reproductive physiology of Paracentrotus lividus lmk: a mesocosm experiment
Source: Front Physiol. 2023 May 23;14:1161852. doi: 10.3389/fphys.2023.1161852 (PMC10242131; doi:10.3389/fphys.2023.1161852)
Supplement: Supplementary file 1 [file DataSheet1.pdf]

Supplementary Material

Morphologic and genic effects of organic pollution on the reproductive physiology of *Paracentrotus lividus* Lmk: a mesocosm experiment

Glaviano F.[1,2], Federico S.[3], Pinto B.[2], Gharbi M.[2], , Russo T.[2], Di Cosmo A.[2], Polese G.[2], Costantini M.[3*], Zupo V.[1*]

[1] Stazione Zoologica Anton Dohrn, Department of Ecosustainable Marine Biotechnology, Ischia Marine Centre, Naples, Italy, Email: francesca.glaviano@szn.it, vzupo@szn.it

[2] Department of Biology, University of Naples Federico II, Complesso Universitario di Monte Sant'Angelo, Via Cinthia 21, 80126 Naples, Italy: brun.pinto@studenti.unina.it, tania.russo@unina.it, anna.dicosmo@unina.it, gianluca.polese@unina.it

[3] Stazione Zoologica Anton Dohrn, Department of Ecosustainable Marine Biotechnology, Via Ammiraglio Ferdinando Acton n. 55, 80133 Napoli, Italy, Email: serena.federico@szn.it, maria.costantini@szn.it

*** Correspondence:**
Maria Costantini; Valerio Zupo
maria.costantini@szn.it; vzupo@szn.it

1 Supplementary Figures and Tables

NAME	ACRONYMS	FUNCTIONS	REFERENCES
**STRESS RESPONSE**			
ADP-ribosylation factor 1	ARF1	An enzyme that catalyzes the trasfer of ADP-rybose from NAD+ to proteins causing their inactivation.	(Esposito et al., 2020)
Cholinesterase	ChE	Acetylcholinesterase, in vertebrates, has a main role in the modulation of neuromuscular impulse transmission. In invertebrates pseudo cholinesterases are pre-eminently represented.	(Cunha et al., 2005)
Citochrome P450 2UI isoform X2	CYP-2UI	This gene encodes for heme-thiolate monooxygenase enzymes, which are involved in stress response	(Goldstone et al., 2006; Albarano et al., 2021)
Glyoxylate reductase hydroxypyruvate reductase	GRHPR	Member of oxidoreductase family that plays a kay role in the reaction of hydroxypiruvate formation starting from D-glycerate.	(Esposito et al., 2020)
Glutathione-S-transferase	GST	This enzyme is expressed in the intestine tissue and it is involved in the response of environmental stresses	(Cunha et al., 2005)
Poly(ADP-ribose) polymerase 1	PARP	Activation of PARP causes the release of AIF, mitochondrial oxidoreductase that induces apoptosis	(Esposito et al., 2020)
Tumor necrosis factor alpha	TNF	Protein that restricts and terminates inflammatory responses through the modulation of the ubiquitination status of central components in NF-κB, IRF3 and apoptosis signaling cascades	(Vereecke et al., 2011)
Heat Shock Protein 70<br>*Heat Shock Protein 60*<br>*Heat Shock Protein 56*	*hsp70*<br>*hsp75*<br>*hsp90*	A family of proteins that are produced by cells in response to exposure to stressful conditions. They were first described in relation to heat shock, but are now known to also be expressed during other stresses including exposure to cold, UV light and during wound healing or tissue remodeling; many members of this group perform chaperone function by stabilizing new proteins to ensure correct folding or by helping to refold proteins that were damaged by the cell stress.	(Marrone et al., 2012)

DNA-methyltransferase 1	*MTase*	A large group of enzymes that all methylate their substrates but can be split into several subclasses based on their structural features; these enzymatic reactions are found in many pathways and are implicated in genetic diseases, cancer, and metabolic diseases.	(Marrone et al., 2012)
Glutamine synthetase	*GS*	An enzyme that plays an essential role in the metabolism of nitrogen by catalyzing the condensation of glutamate and ammonia to form glutamine; the glutamine produced is an essential precursor for purine and pyrimidine synthesis, a modulator of protein turnover or an intermediate for gluconeogenesis and acid-base balance	(Marrone et al., 2012)
Cytochrome b	*cyt b*	A protein found in the mitochondria of eukaryotic cells; it works as part of the electron transport chain and is the main subunit of trans membrane cytochrome bc1 and b6f complexes.	(Marrone et al., 2012)
14-3-3 epsilon protein	*14-3-3ε*	A family of conserved regulatory molecules that are expressed in all eukaryotic cells; they bind a multitude of functionally diverse signaling proteins, including kinases, phosphatases, and transmembrane receptors.	(Marrone et al., 2012)
Sp-Cspe3/7L<br>*caspase-8*	*caspase 3/7*<br>*CASP8*	Protease enzymes playing essential roles in programmed cell death and inflammation; they are named caspases due to their specific cysteine protease activity.	(Romano et al., 2011; Ruocco et al., 2016)
Nuclear factor kappa-light-chain-enhancer of activated B cells	*NF-κB*	A protein complex that controls transcription of DNA, cytokine production and cell survival; it is found in almost all animal cell types and is involved in cellular responses to stimuli such as stress, cytokines, free radicals, heavy metals, ultraviolet irradiation, oxidized LDL, and bacterial or viral antigens.	(Russo et al., 2014a)

Tumor protein p53	*p53*	Tumor suppressors; they bind to DNA and regulate gene expression to prevent mutations of the genome.	(Varrella et al., 2016)
Hypoxia inducible factor 1-alpha	*HIF1A*	A subunit of a heterodimeric transcription factor hypoxia-inducible factor 1 (HIF-1) that is encoded by the HIF1A gene; it is considered as the master transcriptional regulator of cellular and developmental response to hypoxia.	(Varrella et al., 2016)
**SKELETOGENESIS**			
Spicule matrix protein 30 Spicule matrix protein 50	*SM30 SM50*	These proteins direct spicules growth in certain orientations and inhibit growth in others.	(Marrone et al., 2012)
Bone morphogenetic protein 5-7	*BMP5-7*	Promote the oral-aboral ectoderm specification in the sea urchin embryo.	(Marrone et al., 2012)
Nectin	*Nec*	Families of cellular adhesion molecules involved in Ca^{2+}-independent cellular adhesion.	(Marrone et al., 2012)
Univin	*Uni*	Encodes for the Transforming growth factor beta (TGF-β) promoting the interaction of ectodermal cells and the growth of skeleton in sea urchin embryos.	(Marrone et al., 2012)
Pl-p16 Pl-p19	*p16 p19*	Two small acidic proteins involved in the formation of the biomineralized skeleton of sea urchin embryos and adults.	(Costa et al., 2012)
Jun	*Jun*	Transcription factor required for the progression through the G1 phase of the cell cycle.	(Russo et al., 2014b)
**DEVELOPMENT AND DIFFERENTIATION**			
Wnt 5	*Wnt5*	Initiates the specification of the sea urchin posterior ectoderm.	(Varrella et al., 2014)

Wnt 6	*Wnt6*	Activates endoderm in the sea urchin gene regulatory network.	(Varrella et al., 2014)
Nodal	*nodal*	Regulates left-right asymmetry during cleavage and early blastula stages, acting on the right side of the embryo.	(Ruocco et al., 2017)
Transcription factor 4<br>*Transcription factor 7*	*tcf4*<br>*TCF7*	Members of the Tcf/Lef family responsible for the specification of cell fates along the sea urchin animal-vegetal axis, by interacting with β-catenin.	(Ruocco et al., 2017)
Forkhead box protein A Forkhead box protein G Forkhead box protein O	*FOXA*<br>*FoxG*<br>*Foxo*	Members of the Forkhead transcription factors involved in the regulation of embryonic development, cell fate specification, cell differentiation, and morphogenesis.	(Ruocco et al., 2017)
Growth factor indipendent 1	*GFI-1*	Zinc finger transcription factor expressed in the presumptive ciliary band at the mesenchyme blastula stage.	(Ruocco et al., 2017)
One Cut Homeobox 1	*OneCut*	Transcription factor expressed in the early gastrula stage giving rise to the future ciliary band regions and later in the definitive ciliary band of the sea urchin pluteus, including the apical organ.	(Ruocco et al., 2017)
TGF beta-activated kinase	*TAK1*	The major intracellular mediator of the highly conserved TGF beta/BMP signaling pathway implicated in many other different signaling pathways including TNF and interleukin as well as JNK and p38 activities.	(Ruocco et al., 2017)
Vascular endothelial growth factor	*VEGF*	VEGF/VEGFR signaling between ectoderm and the primary mesenchyme cells (PMCs) plays a key role in the positioning and differentiation of these migrating cells during gastrulation and in the morphogenesis of the sea urchin embryonic skeleton.	(Ruocco et al., 2017)
c-Jun N-terminal kinase	*JNK*	Required for cell movements during embryonic development, especially for invagination of the archenteron.	(Ruocco et al., 2017)

Calcium/calmodulin-dependent protein kinase type 1D	*CM-K*	Calcium-binding protein that is present in eggs and involved in the control of nuclear envelope breakdown (NEB) during mitotic division	(Floyd et al., 1986; Baitinger et al., 1990; Albarano et al., 2021)
Camp-responsive element	CREB	Transcription factor that binds certain DNA sequences, named cAMP response elements (CRE), increasing or decreasing the expression of target genes	(Ingham, 1998; Albarano et al., 2021)
Frizzled7	FZ-7	Binding to Wnt6, this receptor is responsible for initiating β-catenin nuclearisation in macromeres at the 5th cleavage, which is necessary for endoderm specification	(Lhomond et al., 2012)
Goosecoid	GOOS	Trascription factor able to induce the expression of two genes, FOXA and Bra , involved in stomodeal formation. It also inhibits the ciliary band formation and the dorsal genes expression.	(Esposito et al., 2020)
Hedgehog	HH	Protein expressed downstream to Brachyury and FoxA in the endomesoderm gene regulatory network during gastrulation that participates to the mesoderm organization	(Walton et al., 2009; Albarano et al., 2021)
Janus kinase	*JAK*	Transcription factor that, binding to the STAT1, plays a kay role in the developmental processes	(Hou et al., 2002; Ito et al., 2004)
**DETOXIFICATION**			
Metallothionein<br>*Metallothionein 4*<br>*Metallothionein 5*<br>*Metallothionein 6*<br>*Metallothionein 7*<br>*Metallothionein 8*	*MT*<br>*MT4*<br>*MT5*<br>*MT6*<br>*MT7*<br>*MT8*	Proteins capable of binding to heavy metals, involved in the transport of heavy metals and cellular detoxification.	(Marrone et al., 2012; Ragusa et al., 2013)
Multi drug resistance protein 1	*MDR1*	ATP-binding cassette protein.	(Varrella et al., 2014)
Catalase	*CAT*	Catalyzes the decomposition of hydrogen peroxide to water and oxygen; it is important in protecting the cell from oxidative damage by reactive oxygen species.	(Varrella et al., 2014)

Supplementary Table 1. Genes name, acronym, function and reference.

Table Analyzed	NH_4 RAS vs Ctrl
Paired t test	
P value	0,0017
P value summary	**
Significantly different (P < 0.05)?	Yes

Supplementary Table 2. Paired t-test to compare concentration trends of NH_4 in the control tanks (open cycle) and experimental tanks (RAS system).

Table Analyzed	NO_2 RAS vs Ctrl
Paired t test	
P value	0,0002
P value summary	***
Significantly different (P < 0.05)?	Yes

Supplementary Table 3. Paired t-test to compare concentration trends of NO2 in the control tanks (open cycle) and experimental tanks (RAS system).

Table Analyzed	NO_3 RAS vs Ctrl
Paired t test	
P value	<0,0001
P value summary	****
Significantly different (P < 0.05)?	Yes

Supplementary Table 4. Paired t-test to compare concentration trends of NO3 in the control tanks (open cycle) and experimental tanks (RAS system).

Table Analyzed	PO_4 RAS vs Ctrl
Paired t test	
P value	0,0566
P value summary	ns
Significantly different (P < 0.05)?	No

Supplementary Table 5. Paired t-test to compare concentration trends of PO4 in the control tanks (open cycle) and experimental tanks (RAS system).

Table Analyzed	T RAS vs Ctrl
Paired t test	

P value	<0,0001
P value summary	****
Significantly different (P < 0.05)?	Yes

Supplementary Table 6. Paired t-test to compare concentration trends of Temperature in the control tanks (open cycle) and experimental tanks (RAS system).

Table Analyzed	pH RAS vs Ctrl
Paired t test	
P value	<0,0001
P value summary	****
Significantly different (P < 0.05)?	Yes

Supplementary Table 7. Paired t-test to compare concentration trends of pH in the control tanks (open cycle) and experimental tanks (RAS system).

Table Analyzed	O_2 RAS vs Ctrl
Paired t test	
P value	<0,0001
P value summary	****

Significantly different (P < 0.05)?	Yes

Supplementary Table 8. Paired t-test to compare concentration trends of O2 in the control tanks (open cycle) and experimental tanks (RAS system).

Table Analyzed	Salinity RAS vs Ctrl
Paired t test	
P value	<0,0001
P value summary	****
Significantly different (P < 0.05)?	Yes

Supplementary Table 9. Paired t-test to compare concentration trends of Salinity in the control tanks (open cycle) and experimental tanks (RAS system).

Table Analyzed	Mortality
Unpaired t test	
P value	0,0099
P value summary	**
Significantly different (P < 0.05)?	Yes

Supplementary Table 10. Unaired t-test to compare Mortality in the control tanks (open cycle) and experimental tanks (RAS system).

A

Table Analyzed	Delayed larvae
Unpaired t test	
P value	0,0133
P value summary	*
Significantly different (P < 0.05)?	Yes

B

Table Analyzed	Normal Plutea
Unpaired t test	
P value	0,0133
P value summary	*
Significantly different (P < 0.05)?	Yes

Supplementary Table 11. A) Unaired t-test to compare results of larval development and abnormalities produced at 48 hpf in the fertilization tests from reproducers coming from the control tanks (open cycle) and experimental tanks (RAS system). As delayed larvae we considered all the embryos arrested at the blastula and gastrula stage and the prisms; B) Unaired t-test to compare results of normal larvae produced at 48 hpf in the fertilization tests from reproducers coming from the control tanks (open cycle) and experimental tanks (RAS system).

Table Analyzed	Gonadosomatic index
Unpaired t test	
P value	0,6852
P value summary	ns
Significantly different (P < 0.05)?	No

Supplementary Table 12. Unaired t-test to compare Gonadosomatic index from animals reared in the control tanks (open cycle) and experimental tanks (RAS system).

2 References

Albarano, L., Zupo, V., Guida, M., Libralato, G., Caramiello, D., Ruocco, N., et al. (2021). PAHs and PCBs Affect Functionally Intercorrelated Genes in the Sea Urchin *Paracentrotus lividus* Embryos. *Int J Mol Sci* 22. doi: 10.3390/ijms222212498.

Baitinger, C., Alderton, J., Poenie, M., Schulman, H., and Steinhardt, R. A. (1990). Multifunctional Ca2+/calmodulin-dependent protein kinase is necessary for nuclear envelope breakdown. *J Cell Biol* 111, 1763. doi: 10.1083/JCB.111.5.1763.

Costa, C., Karakostis, K., Zito, F., and Matranga, V. (2012). Phylogenetic analysis and expression patterns of p16 and p19 in *Paracentrotus lividus* embryos. *Dev Genes Evol* 222. doi: 10.1007/s00427-012-0405-9.

Cunha, I., García, L. M., and Guilhermino, L. (2005). Sea-urchin (Paracentrotus lividus) glutathione S-transferases and cholinesterase activities as biomarkers of environmental contamination. *J Environ Monit* 7, 288–294. doi: 10.1039/B414773A.

Esposito, R., Ruocco, N., Albarano, L., Ianora, A., Manfra, L., Libralato, G., et al. (2020). Combined effects of diatom-derived oxylipins on the sea urchin *Paracentrotus lividus*. *Int J Mol Sci* 21, 719.

Floyd, E. E., Gong, Z., Brandhorst, B. P., and Klein, W. H. (1986). Calmodulin gene expression during sea urchin development: persistence of a prevalent maternal protein. *Dev Biol* 113, 501–511. doi: 10.1016/0012-1606(86)90185-5.

Goldstone, J. v., Hamdoun, A., Cole, B. J., Howard-Ashby, M., Nebert, D. W., Scally, M., et al. (2006). The chemical defensome: environmental sensing and response genes in the *Strongylocentrotus purpuratus* genome. *Dev Biol* 300, 366–384. doi: 10.1016/J.YDBIO.2006.08.066.

Hou, S. X., Zheng, Z., Chen, X., and Perrimon, N. (2002). The JAK/STAT pathway in model organisms: Emerging roles in cell movement. *Dev Cell* 3. doi: 10.1016/S1534-5807(02)00376-3.

Ingham, P. W. (1998). Transducing Hedgehog: the story so far. *EMBO J* 17, 3505–3511. doi: 10.1093/EMBOJ/17.13.3505.

Ito, M., Nakasato, M., Suzuki, T., Sakai, S., Nagata, M., and Aoki, F. (2004). Localization of Janus kinase 2 to the nuclei of mature oocytes and early cleavage stage mouse embryos. *Biol Reprod* 71. doi: 10.1095/biolreprod.103.023226.

Lhomond, G., McClay, D. R., Gache, C., and Croce, J. C. (2012). Frizzled1/2/7 signaling directs β-catenin nuclearisation and initiates endoderm specification in macromeres during sea urchin embryogenesis. *Development* 139. doi: 10.1242/dev.072215.

Marrone, V., Piscopo, M., Romano, G., Ianora, A., Palumbo, A., and Costantini, M. (2012). Defensome against Toxic Diatom Aldehydes in the Sea Urchin *Paracentrotus lividus*. *PLoS One* 7, e31750. doi: 10.1371/JOURNAL.PONE.0031750.

Ragusa, M. A., Costa, S., Gianguzza, M., Roccheri, M. C., and Gianguzza, F. (2013). Effects of cadmium exposure on sea urchin development assessed by SSH and RT-qPCR: Metallothionein genes and their differential induction. *Mol Biol Rep* 40, 2157–2167.

Romano, G., Costantini, M., Buttino, I., Ianora, A., and Palumbo, A. (2011). Nitric Oxide Mediates the Stress Response Induced by Diatom Aldehydes in the Sea Urchin *Paracentrotus lividus*. *PLoS One* 6, e25980. doi: 10.1371/JOURNAL.PONE.0025980.

Ruocco, N., Costantini, M., and Santella, L. (2016). New insights into negative effects of lithium on sea urchin *Paracentrotus lividus* embryos. *Sci Rep* 6, 1–12.

Ruocco, N., Costantini, S., Zupo, V., Romano, G., Ianora, A., Fontana, A., et al. (2017). High-quality RNA extraction from the sea urchin *Paracentrotus lividus* embryos. *PLoS One* 12, e0172171.

Russo, R., Pinsino, A., Costa, C., Bonaventura, R., Matranga, V., and Zito, F. (2014a). The newly characterized Pl□jun is specifically expressed in skeletogenic cells of the Paracentrotus lividus sea urchin embryo. *FEBS J* 281, 3828–3843.

Russo, R., Pinsino, A., Costa, C., Bonaventura, R., Matranga, V., and Zito, F. (2014b). The newly characterized Pl□jun is specifically expressed in skeletogenic cells of the *Paracentrotus lividus* sea urchin embryo. *FEBS J* 281, 3828–3843.

Varrella, S., Romano, G., Ianora, A., Bentley, M. G., Ruocco, N., and Costantini, M. (2014). Molecular response to toxic diatom-derived aldehydes in the sea urchin *Paracentrotus lividus*. *Mar Drugs* 12, 2089–2113.

Varrella, S., Romano, G., Ruocco, N., Ianora, A., Bentley, M. G., and Costantini, M. (2016). First morphological and molecular evidence of the negative impact of diatom-derived hydroxyacids on the sea urchin *Paracentrotus lividus*. *Toxicological Sciences* 151, 419–433.

Vereecke, L., Beyaert, R., and van Loo, G. (2011). Genetic relationships between A20/TNFAIP3, chronic inflammation and autoimmune disease. in *Biochemical Society Transactions* doi: 10.1042/BST0391086.

Walton, K. D., Warner, J., Hertzler, P. H., and McClay, D. R. (2009). Hedgehog signaling patterns mesoderm in the sea urchin. *Dev Biol* 331, 26–37. doi: 10.1016/J.YDBIO.2009.04.018.
